# Supplementary material for: Intermediate excited state relaxation dynamics of boron vacancy spin defects in hexagonal boron nitride
Source: Sci Adv. 2026 Feb 25;12(9):eaea0109. doi: 10.1126/sciadv.aea0109 (PMC12935034; doi:10.1126/sciadv.aea0109)
Supplement: Supplementary file 1 — Supplementary Text Figs. S1 to S8 Legend for code S1 Legend for data S1 References [file sciadv.aea0109_sm.pdf]

Science Advances
AAAS

Supplementary Materials for

Intermediate excited state relaxation dynamics of boron vacancy spin defects in hexagonal boron nitride

Paul Konrad *et al.*

Corresponding author: Vladimir Dyakonov, vladimir.dyakonov@uni-wuerzburg.de

Sci. Adv. **12**, eaea0109 (2026)
DOI: 10.1126/sciadv.aea0109

The PDF file includes:

Supplementary Text
Figs. S1 to S8
Legend for code S1
Legend for data S1
References

Other Supplementary Material for this manuscript includes the following:

Code S1
Data S1

Supplementary Text

Sensitivity Estimation for Spin Packet

We estimate the spin-projection limited sensitivity $\delta B \approx \frac{h}{g_e \mu_B R \sqrt{\eta}\sqrt{N\tau T_2^*}}$ given by (20,41) for our current setup. Here, h is the Planck constant, g_e is the gyromagnetic ration, μ_B is the Bohr magneton, R is the ODMR contrast, η is the collection efficiency, N is the number of addressed defects, τ is the interrogation time and T_2^* is the dephasing time. Unfortunately, we have no direct measure of the absolute number of spins we address since classical EPR methods fail due to the small sample volume (and inhomogeneity). A recent own publication allows us however to estimate the number of simultaneously addressed spins via Raman features (42) and we can assume to address $5.8 \cdot 10^{18} \frac{\text{defects}}{\text{cm}^3} * \pi * 1.22 * \left(\frac{473\text{nm}}{2\,NA}\right)^2 * 100\text{nm}$, with a diffraction limited spot of size $\approx 1.22 * \frac{473\text{nm}}{NA}$, a numerical aperture of the objective of $NA = 0.9$ and a flake thickness of 100nm. These assumptions yield an estimate of $2 \cdot 10^5$ addressed defects. For measurement time we can also take $\tau = 1ns$. The collection efficiency η can be estimated to be significantly higher than for the previous publication by Gottscholl et al., since the numerical aperture changed from 0.3 to 0.9. Nevertheless, for comparison and since a lot more factors determine the collection efficiency, we keep the assumption of the efficiency to be 1%. The Contrast of the ODMR measurement also significantly increased to 2.5% as shown in Fig. 1 of the main text. The decoherence time T_2^* can be estimated by the envelope of the Rabi oscillation to 56ns (see main text). With these assumptions, one obtains a minimal detectable magnetic field of $\delta B \approx 100\text{nT}/\sqrt{\text{Hz}}$ which is in the same order of magnitude as from Gottscholl et al (20).

5-Level Rate Model:

In this work we use a simplified 5-level model for the V_B^- spin system shown in Fig. 1(b) of the main text. The system is assumed to be split into a triplet sub-system and a singlet sub-system with a single metastable intermediate state (IS). The triplet sub-system has a non-degenerate ground and excited state (GS, ES) where the energy levels are associated with the projections of the spin $m_S = 0$ and $m_s = \pm 1$ (combined into one level) are split by the zero-field splitting of D_{GS} = 3.49 GHz (*14*) and D_{ES} = 2.09 GHz (*29*), respectively. Electrons can be excited by visible laser light with a relaxation path emitting photons in the near infrared around 850 nm (*14, 34*). From the ES, electrons can undergo spin-conserving optical relaxation or spin-selective and non-conserving relaxation via an intermediate state (*28*). The spin-selective relaxation enables optically detected magnetic resonance (ODMR) and the non-conserving property together with favored relaxation into the $m_s = 0$ ground state leads to spin polarization which also allows for coherent control (*18*).

Simulation code

The Python code provided as a separate file is used to simulate the PL response inside of the 5-level rate model. The excitation rate can be modified to account for the smearing of the laser power. Rising and settling of the excitation rate is simulated smoothly by a hyperbolic function. The PL of the 5-level model is simulated by the code submitted with this manuscript.

For simulations, the starting values for rates are adapted from literature:

$k_e = 27\ \mu s^{-1}$

$k_r = 90.9\ ms^{-1}$ (*28*)

$\gamma_0 = 820\ \mu s^{-1}$ (*31*)

$\gamma_1 = 1.89\ ns^{-1}$ (*31*)

$\kappa_0 = 31.2\ \mu s^{-1}$

$\kappa_1 = 1/3 \cdot \kappa_0$ (*29, 31*)

The value of κ_0 is determined by the relation $\kappa_1 = 1/3 \cdot \kappa_0$ and the IS lifetime $T_{IS} = 1/(\kappa_0+\kappa_1) = 24.0(3)$ ns. The values of γ_0 and γ_1 were determined in (*31*) using pulsed laser excitation. This is a method that we expect to be highly reliable. It is noteworthy that (*29*) used the same approach with shorter laser pulses, hence expected better instrument response function. However, as Clua-Provost et al. showed with 18 samples, there is slight deviation from sample to sample. We therefore use the statistically more robust value from (*31*).

The relation $\kappa_1 = 1/3 \cdot \kappa_0$ is well agreed upon between Clua-Provost et al. and Baber et al., although a different notation is used (*29, 31*). In the first, κ_1 is used for the relaxation rate from the intermediate state into a single ground state level (assumed to be $m_s = 1$), while the latter and the main text use κ_1 as the overall rate with which the system relaxes back into the ground state $m_s = \pm 1$ sub-manifold. It is then implied by Clua-Provost et al. that this rate is the same for the $m_s = -1$ state, hence the given total lifetime of the IS of $(\kappa_0 + 2 \cdot \kappa_1) = T_{IS}$

The value k_r is not known experimentally. The only estimation that is available is from calculations by Reimers et al. (*28*). Nevertheless, the exact value of this rate is not decisive for the dynamics. Most relevant is that it is significantly smaller than all the other relaxation rates in the system.

The value for k_e is estimated manually by simulation to gain good fitting of the experimental data.

Transient Photoluminescence

Transient PL for various dark periods was conducted in one long pulse train. The sequence is uploaded to the Swabian instruments Pulse Streamer 8/2 device and repeated continuously. PL data is recorded by a combination of an Excelitas single photon detector and the time-correlated single-photon counting module Time Tagger 20 from Swabian instruments. The data is averaged until the signal-to-noise ratio is satisfying.

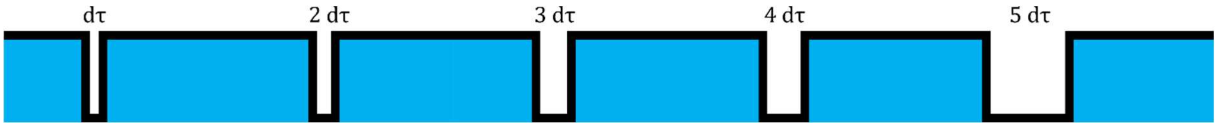

Fig. S1: Pulse train schematic for transient PL measurements.

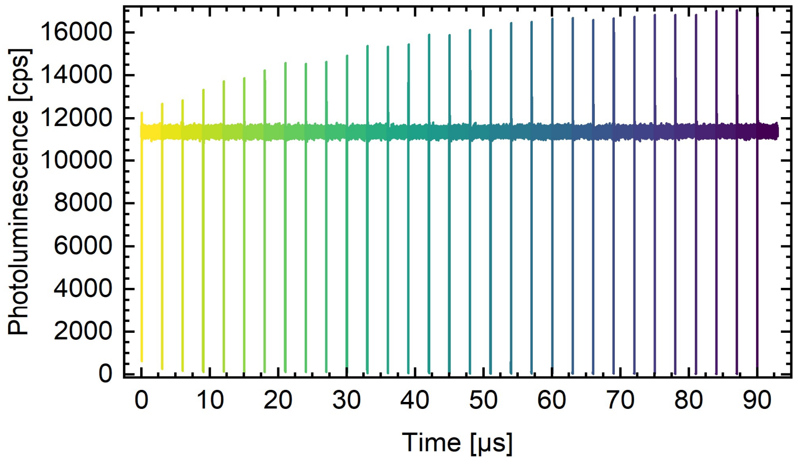

Fig. S2: Transient PL trace with increasing dark periods measured as one continuous pulse train. The dark periods (not resolved in this scale) increase from left to right. The increase of the PL overshoot due to repopulation of the GS can clearly be seen. The data reliably shares a common stable state with a shared level in PL intensity.

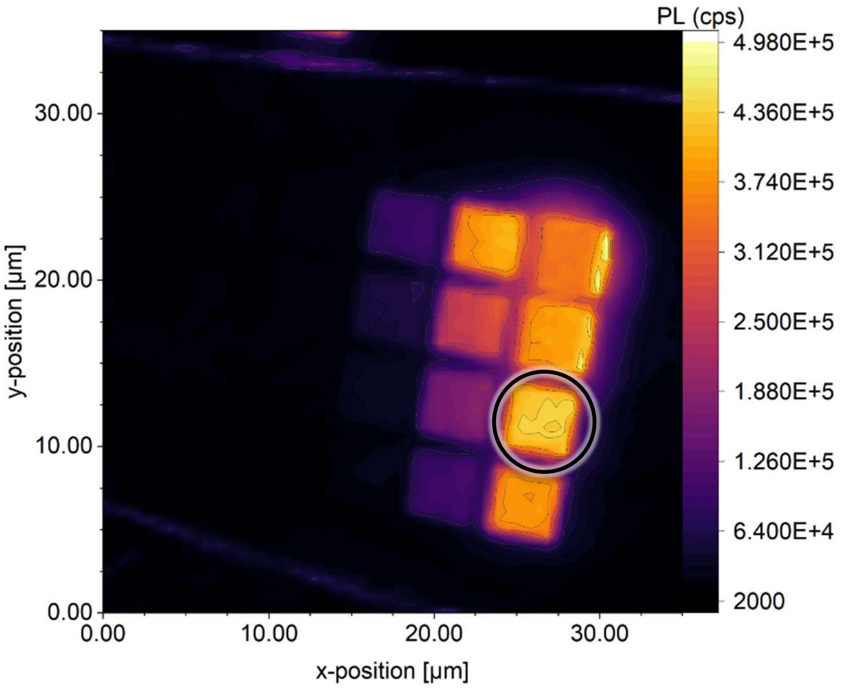

Fig. S3: Laterally resolved PL of the hBN sample studied in this work. Measurements were conducted on the irradiated area with the highest PL intensity, highlighted by a circle.

Dependence of V_B^- photoluminescence on laser power

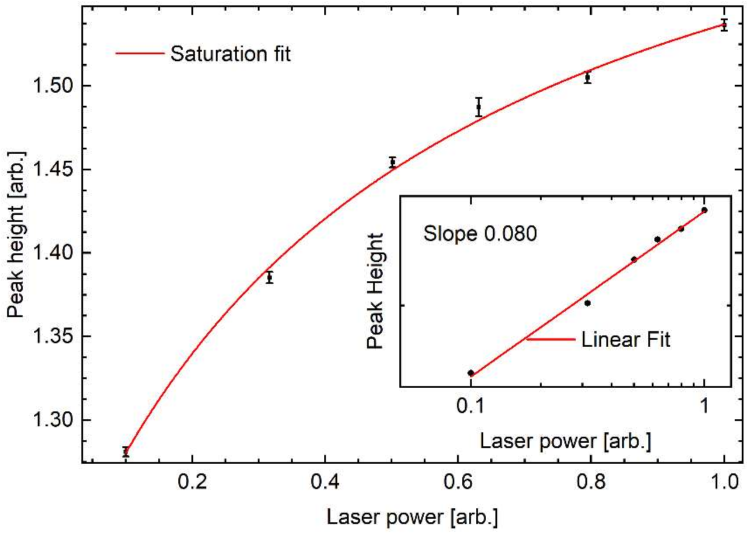

Fig. S4: Peak height of the PL overshoot depending on the laser power for a dark period of 150 ns. As can be seen, the peak value begins to saturate, indicating excitation of a large portion of the GS population.

Figure S4 shows the dependence of the maximum overshoot peak height on the laser power for the transients shown in Fig. 2 of the main text. The onset of saturation can be observed, and the following model is fitted to the data:

$$y = y_0 + \frac{h_{\text{sat}}}{1+\frac{P_{½}}{P}}$$

The fit yields h_{sat}=0.52 and $P_{½}$=0.54.

Additional simulations

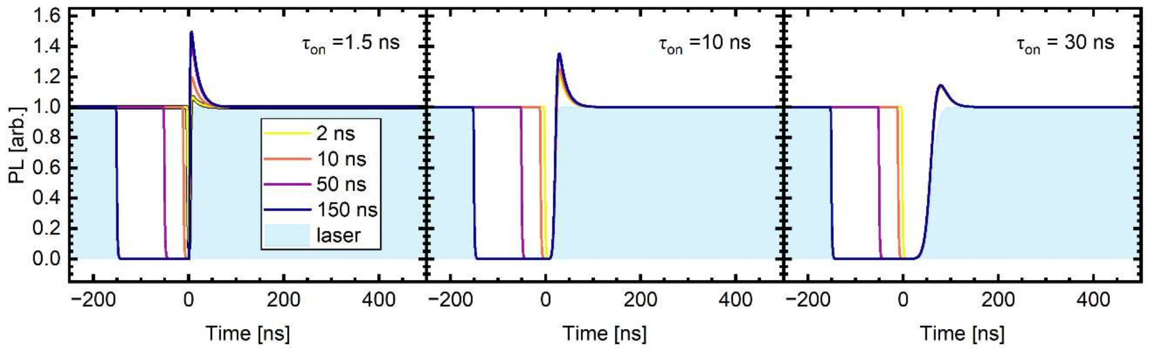

Fig. S5: Simulated PL transients for various dark periods. The simulation is repeated for various values of smearing of the rising and falling flanks of the laser excitation. The laser excitation is shown as the blue shaded area for a dark period of 150 ns. The overshoot decreases significantly with softer turn-on of the laser and the dependence on the dark period becomes insignificant.

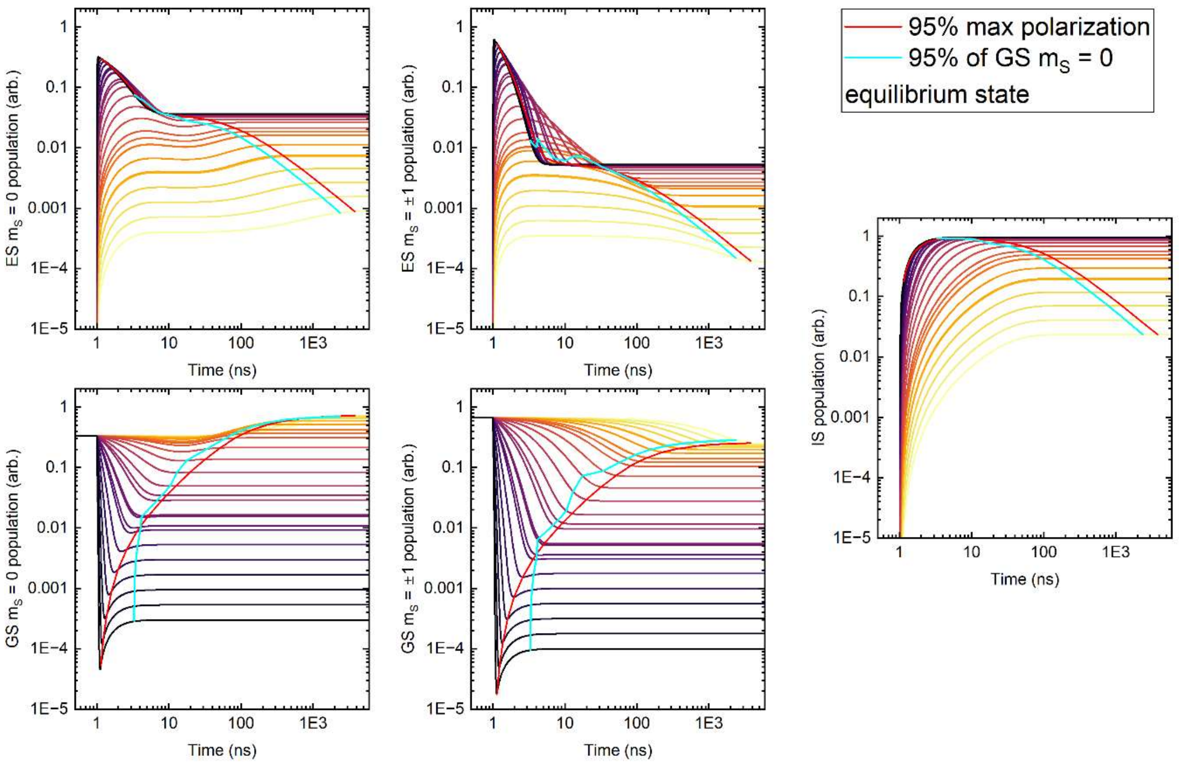

Fig. S6: Simulated populations of all sub states for various excitation rates in units of total population. The overlying lines are for two thresholds, namely for equilibrium in the GS m_S = 0 state (cyan line) that was also used for Fig. 3c and for the time required to gain 95% of the maximum spin polarization (75%, red line).

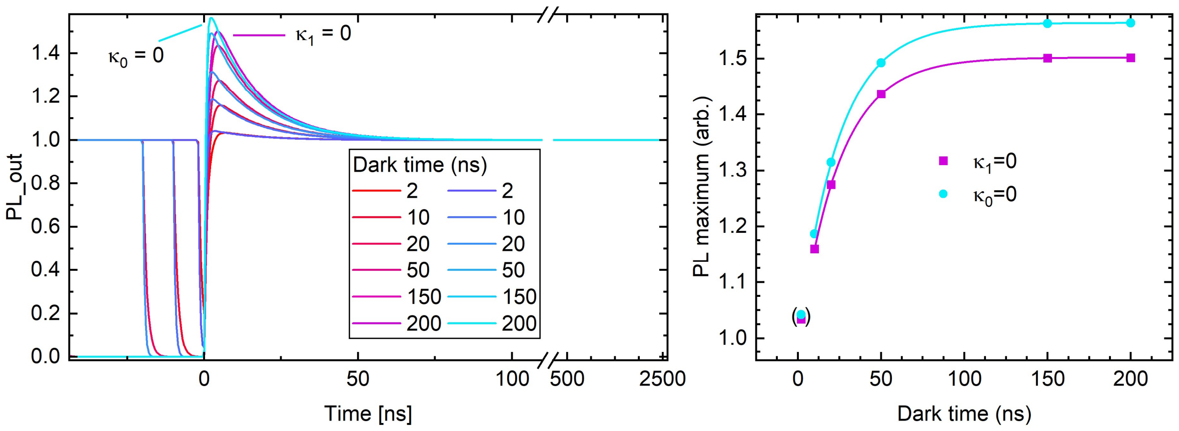

Fig S7: Influence of the ratio κ_1/κ_0 on transients. The simulations were conducted for the extreme cases of $\kappa_1 = 0$ and $\kappa_0 = 0$. Left: Transients with the given κ set to 0. The κ ratio is not experimentally accessible with our method. The transients do not differ significantly and therefore do not change the interpretation of our data. Right: Calculated PL overshoots for the respective κ set to 0. The calculated PL maximum follows the same exponential trend with the same IS lifetime. In the fits, the first data point for 2ns dark period is omitted since the relaxation rates are too slow to deplete the ES in time, resulting in an expected deviation from exponential growth.

Laser response measured by the photoluminescence of silicon substrates

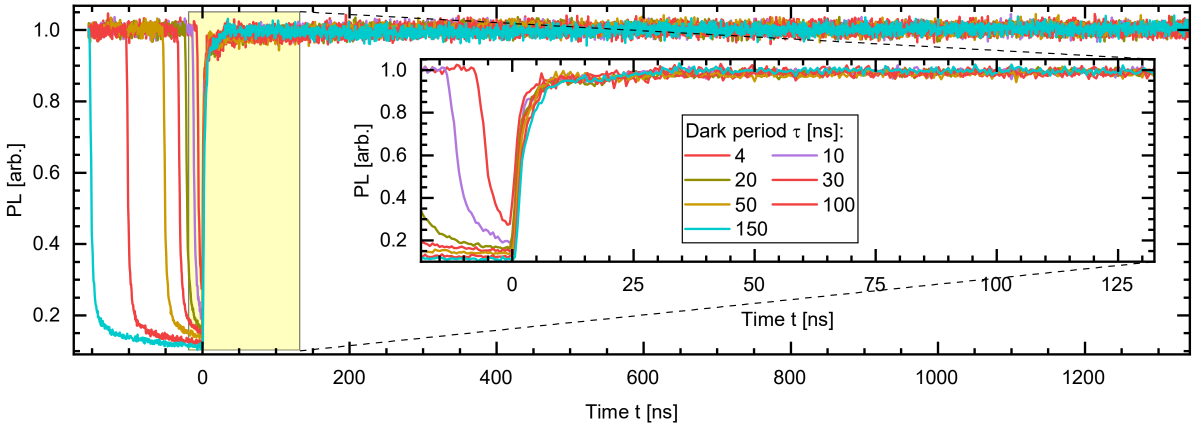

Fig. S8: Transient PL of the silicon substrate for several dark periods. No PL overshoot is observed. This shows that the PL overshoot of V_B^- discussed in this paper is not an artifact of the laser on/off modulation and / or instrument response function.

Code S1

Python code for simulating the transient photoluminescence with an example for a pulsed laser excitation and dark period of 150ns. Requires the packages numpy and matplotlib

Data S1

Data shown in the figure plots of the main text. Each subfigure is listed as a separate sheet. Data in subfigures containing multiple datasets is labeled column-wise. The most left column of each data block contains x-axis values.

REFERENCES

1. E. Knill, Physics: Quantum computing. *Nature* **463**, 441–443 (2010).

2. C. L. Degen, F. Reinhard, P. Cappellaro, Quantum sensing. *Rev. Mod. Phys.* **89**, 035002 (2017).

3. J. M. Taylor, P. Cappellaro, L. Childress, L. Jiang, D. Budker, P. R. Hemmer, A. Yacoby, R. Walsworth, M. D. Lukin, High-sensitivity diamond magnetometer with nanoscale resolution. *Nat. Phys.* **4**, 810–816 (2008).

4. G. Balasubramanian, I. Y. Chan, R. Kolesov, M. Al-Hmoud, J. Tisler, C. Shin, C. Kim, A. Wojcik, P. R. Hemmer, A. Krueger, T. Hanke, A. Leitenstorfer, R. Bratschitsch, F. Jelezko, J. Wrachtrup, Nanoscale imaging magnetometry with diamond spins under ambient conditions. *Nature* **455**, 648–651 (2008).

5. V. M. Acosta, E. Bauch, M. P. Ledbetter, A. Waxman, L.-S. Bouchard, D. Budker, Temperature dependence of the nitrogen-vacancy magnetic resonance in diamond. *Phys. Rev. Lett.* **104**, 70801 (2010).

6. M. W. Doherty, V. M. Acosta, A. Jarmola, M. S. J. Barson, N. B. Manson, D. Budker, L. C. L. Hollenberg, Temperature shifts of the resonances of the NV^- center in diamond. *Phys. Rev. B* **90**, 041201 (2014).

7. H. Kraus, V. A. Soltamov, F. Fuchs, D. Simin, A. Sperlich, P. G. Baranov, G. V. Astakhov, V. Dyakonov, Magnetic field and temperature sensing with atomic-scale spin defects in silicon carbide. *Sci. Rep.* **4**, 5303 (2014).

8. C. R. Dean, A. F. Young, I. Meric, C. Lee, L. Wang, S. Sorgenfrei, K. Watanabe, T. Taniguchi, P. Kim, K. L. Shepard, J. Hone, Boron nitride substrates for high-quality graphene electronics. *Nat. Nanotechnol.* **5**, 722–726 (2010).

9. T. Georgiou, R. Jalil, B. D. Belle, L. Britnell, R. V. Gorbachev, S. V. Morozov, Y.-J. Kim, A. Gholinia, S. J. Haigh, O. Makarovsky, L. Eaves, L. A. Ponomarenko, A. K. Geim, K. S. Novoselov, A. Mishchenko, Vertical field-effect transistor based on graphene-WS_2 heterostructures for flexible and transparent electronics. *Nat. Nanotechnol.* **8**, 100–103 (2013).

10. A. V. Kretinin, Y. Cao, J. S. Tu, G. L. Yu, R. Jalil, K. S. Novoselov, S. J. Haigh, A. Gholinia, A. Mishchenko, M. Lozada, T. Georgiou, C. R. Woods, F. Withers, P. Blake, G. Eda, A. Wirsig, C. Hucho, K. Watanabe, T. Taniguchi, A. K. Geim, R. V. Gorbachev, Electronic properties of graphene encapsulated with different two-dimensional atomic crystals. *Nano Lett.* **14**, 3270–3276 (2014).

11. T. T. Tran, K. Bray, M. J. Ford, M. Toth, I. Aharonovich, Quantum emission from hexagonal boron nitride monolayers. *Nat. Nanotechnol.* **11**, 37–41 (2016).

12. G. Grosso, H. Moon, B. Lienhard, S. Ali, D. K. Efetov, M. M. Furchi, P. Jarillo-Herrero, M. J. Ford, I. Aharonovich, D. Englund, Tunable and high-purity room temperature single-photon emission from atomic defects in hexagonal boron nitride. *Nat. Commun.* **8**, 705 (2017).

13. M. Abdi, J.-P. Chou, A. Gali, M. B. Plenio, Color centers in hexagonal boron nitride monolayers: A group theory and ab initio analysis. *ACS Photonics* **5**, 1967–1976 (2018).

14. A. Gottscholl, M. Kianinia, V. Soltamov, S. Orlinskii, G. Mamin, C. Bradac, C. Kasper, K. Krambrock, A. Sperlich, M. Toth, I. Aharonovich, V. Dyakonov, Initialization and read-out of intrinsic spin defects in a van der Waals crystal at room temperature. *Nat. Mater.* **19**, 540–545 (2020).

15. N. Chejanovsky, A. Mukherjee, J. Geng, Y.-C. Chen, Y. Kim, A. Denisenko, A. Finkler, T. Taniguchi, K. Watanabe, D. B. R. Dasari, P. Auburger, A. Gali, J. H. Smet, J. Wrachtrup, Single-spin resonance in a van der Waals embedded paramagnetic defect. *Nat. Mater.* **20**, 1079–1084 (2021).

16. H. L. Stern, Q. Gu, J. Jarman, S. Eizagirre Barker, N. Mendelson, D. Chugh, S. Schott, H. H. Tan, H. Sirringhaus, I. Aharonovich, M. Atatüre, Room-temperature optically detected magnetic resonance of single defects in hexagonal boron nitride. *Nat. Commun.* **13**, 618 (2022).

17. V. Ivády, G. Barcza, G. Thiering, S. Li, H. Hamdi, J.-P. Chou, Ö. Legeza, A. Gali, Ab initio theory of the negatively charged boron vacancy qubit in hexagonal boron nitride. *npj Comput. Mater.* **6**, 41 (2020).

18. A. Gottscholl, M. Diez, V. Soltamov, C. Kasper, A. Sperlich, M. Kianinia, C. Bradac, I. Aharonovich, V. Dyakonov, Room temperature coherent control of spin defects in hexagonal boron nitride. *Sci. Adv.* **7**, eabf3630 (2021).

19. X. Gao, S. Vaidya, K. Li, P. Ju, B. Jiang, Z. Xu, A. E. L. Allcca, K. Shen, T. Taniguchi, K. Watanabe, S. A. Bhave, Y. P. Chen, Y. Ping, T. Li, Nuclear spin polarization and control in hexagonal boron nitride. *Nat. Mater.* **21**, 1024–1028 (2022).

20. A. Gottscholl, M. Diez, V. Soltamov, C. Kasper, D. Krauße, A. Sperlich, M. Kianinia, C. Bradac, I. Aharonovich, V. Dyakonov, Spin defects in hBN as promising temperature, pressure and magnetic field quantum sensors. *Nat. Commun.* **12**, 4480 (2021).

21. W. Liu, Z.-P. Li, Y.-Z. Yang, S. Yu, Y. Meng, Z.-A. Wang, Z.-C. Li, N.-J. Guo, F.-F. Yan, Q. Li, J.-F. Wang, J.-S. Xu, Y.-T. Wang, J.-S. Tang, C.-F. Li, G. Guo, Temperature-dependent energy-level shifts of spin defects in hexagonal boron nitride. *ACS Photonics* **8**, 1889–1895 (2021).

22. J.-P. Tetienne, Quantum sensors go flat. *Nat. Phys.* **17**, 1074–1075 (2021).

23. M. Huang, J. Zhou, H. L. Di Chen, N. J. McLaughlin, S. Li, M. Alghamdi, D. Djugba, J. Shi, H. Wang, C. R. Du, Wide field imaging of van der Waals ferromagnet Fe_3GeTe_2 by spin defects in hexagonal boron nitride. *Nat. Commun.* **13**, 5369 (2022).

24. A. J. Healey, S. C. Scholten, T. Yang, J. A. Scott, G. J. Abrahams, I. O. Robertson, X. F. Hou, Y. F. Guo, S. Rahman, Y. Lu, M. Kianinia, I. Aharonovich, J.-P. Tetienne, Quantum microscopy with van der Waals heterostructures. *Nat. Phys.* **19**, 87–91 (2023).

25. R. Rizzato, M. Schalk, S. Mohr, J. C. Hermann, J. P. Leibold, F. Bruckmaier, G. Salvitti, C. Qian, P. Ji, G. V. Astakhov, U. Kentsch, M. Helm, A. V. Stier, J. J. Finley, D. B. Bucher,

Extending the coherence of spin defects in hBN enables advanced qubit control and quantum sensing. *Nat. Commun.* **14**, 5089 (2023).

26. C. J. Patrickson, S. Baber, B. B. Gaál, A. J. Ramsay, I. J. Luxmoore, High frequency magnetometry with an ensemble of spin qubits in hexagonal boron nitride. *Npj Quantum Inf.* **10**, 5 (2024).

27. A. Durand, T. Clua-Provost, F. Fabre, P. Kumar, J. Li, J. H. Edgar, P. Udvarhelyi, A. Gali, X. Marie, C. Robert, J. M. Gérard, B. Gil, G. Cassabois, V. Jacques, Optically active spin defects in few-layer thick hexagonal boron nitride. *Phys. Rev. Lett.* **131**, 116902 (2023).

28. J. R. Reimers, J. Shen, M. Kianinia, C. Bradac, I. Aharonovich, M. J. Ford, P. Piecuch, Photoluminescence, photophysics, and photochemistry of the VB− defect in hexagonal boron nitride. *Phys. Rev. B* **102**, 144105 (2020).

29. S. Baber, R. N. E. Malein, P. Khatri, P. S. Keatley, S. Guo, F. Withers, A. J. Ramsay, I. J. Luxmoore, Excited state spectroscopy of boron vacancy defects in hexagonal boron nitride using time-resolved optically detected magnetic resonance. *Nano Lett.* **22**, 461–467 (2022).

30. B. Whitefield, M. Toth, I. Aharonovich, J.-P. Tetienne, M. Kianinia, Magnetic field sensitivity optimization of negatively charged boron vacancy defects in hBN. *Adv. Quantum Tech.* **8**, 2300118 (2025).

31. T. Clua-Provost, Z. Mu, A. Durand, C. Schrader, J. Happacher, J. Bocquel, P. Maletinsky, J. Fraunié, X. Marie, C. Robert, G. Seine, E. Janzen, J. H. Edgar, B. Gil, G. Cassabois, V. Jacques, Spin-dependent photodynamics of boron-vacancy centers in hexagonal boron nitride. *Phys. Rev. B* **110**, 014104 (2024).

32. W. Lee, V. S. Liu, Z. Zhang, S. Kim, R. Gong, Du Xinyi, K. Pham, T. Poirier, Z. Hao, J. H. Edgar, P. Kim, zu Chong, E. J. Davis, N. Y. Yao, Intrinsic high-fidelity spin polarization of charged vacancies in hexagonal boron nitride. arXiv:2406.11953v1 [quant-ph] (2024).

33. X. Gao, S. Pandey, M. Kianinia, J. Ahn, P. Ju, I. Aharonovich, N. Shivaram, T. Li, Femtosecond laser writing of spin defects in hexagonal boron nitride. *ACS Photonics* **8**, 994–1000 (2021).

34. M. Kianinia, S. White, J. E. Fröch, C. Bradac, I. Aharonovich, Generation of spin defects in hexagonal boron nitride. *ACS Photonics* **7**, 2147–2152 (2020).

35. F. F. Murzakhanov, B. V. Yavkin, G. V. Mamin, S. B. Orlinskii, I. E. Mumdzhi, I. N. Gracheva, B. F. Gabbasov, A. N. Smirnov, V. Y. Davydov, V. A. Soltamov, Creation of negatively charged boron vacancies in hexagonal boron nitride crystal by electron irradiation and mechanism of inhomogeneous broadening of boron vacancy-related spin resonance lines. *Nanomaterials* **11**, 1373 (2021).

36. N. B. Manson, J. P. Harrison, M. J. Sellars, Nitrogen-vacancy center in diamond: Model of the electronic structure and associated dynamics. *Phys. Rev. B* **74**, 104303 (2006).

37. Z. Mu, H. Cai, D. Chen, Z. Jiang, S. Ru, X. Lyu, X. Liu, I. Aharonovich, W. Gao, Excited-state optically detected magnetic resonance of spin defects in hexagonal boron nitride. *Phys. Rev. Lett.* **128**, 216402 (2022).

38. N. Mendelson, R. Ritika, M. Kianinia, J. Scott, S. Kim, J. E. Fröch, C. Gazzana, M. Westerhausen, L. Xiao, S. S. Mohajerani, S. Strauf, M. Toth, I. Aharonovich, Z.-Q. Xu, Coupling spin defects in a layered material to nanoscale plasmonic cavities. *Adv. Mater.* **34**, e2106046 (2022).

39. J. F. Barry, J. M. Schloss, E. Bauch, M. J. Turner, C. A. Hart, L. M. Pham, R. L. Walsworth, Sensitivity optimization for NV-diamond magnetometry. *Rev. Mod. Phys.* **92**, 015004 (2020).

40. D. Budker, M. Romalis, Optical magnetometry. *Nat. Phys.* **3**, 227–234 (2007).

41. V. M. Acosta, E. Bauch, M. P. Ledbetter, C. Santori, K.-M. C. Fu, P. E. Barclay, R. G. Beausoleil, H. Linget, J. F. Roch, F. Treussart, S. Chemerisov, W. Gawlik, D. Budker, Diamonds with a high density of nitrogen-vacancy centers for magnetometry applications. *Phys. Rev. B* **80**, 115202 (2009).

42. A. Patra, P. Konrad, A. Sperlich, T. Biktagirov, W. G. Schmidt, L. Spencer, I. Aharonovich, S. Höfling, V. Dyakonov, Quantifying spin defect density in hBN via Raman and photoluminescence analysis. *Adv. Funct. Mater.*, e17851 (2025).
